# Outcomes of isolated ostial LAD PCI versus multivessel PCI including ostial LAD lesions

**DOI:** 10.1016/j.ahjo.2026.100818

**Published:** 2026-06-20

**Authors:** Eleni Ntantou, William Camilleri, Joost Daemen, Roberto Diletti, Jeroen Wilschut, Isabella Kardys, Rutger-Jan Nuis, Nicolas M. van Mieghem, Wijnand K. den Dekker

**Affiliations:** a Department of Cardiology, Cardiovascular Institute, Thoraxcenter, Erasmus MC, University Medical Center, Rotterdam, The Netherlands

**Keywords:** Ostial left anterior descending artery, Multivessel coronary artery disease, Major adverse cardiovascular events, Percutaneous coronary intervention

## Abstract

**Background:**

Ostial lesions of the left anterior descending (LAD) coronary artery are critical targets for revascularization due to their role in supplying a large portion of the myocardium, and are often accompanied by multivessel disease, complicating treatment planning and prognosis.

**Methods:**

This was a retrospective, single-center study. Patients with ostial LAD PCI isolated or in conjunction with PCI in another vessel were included and were grouped into isolated ostial LAD PCI (*n* = 276) or multivessel PCI including ostial LAD PCI (*n* = 218). The primary endpoint was major adverse cardiovascular events (MACE) defined by a composite of all-cause mortality, myocardial infarction, stroke, or unplanned repeat revascularization.

**Results:**

Participants undergoing multivessel PCI were significantly older, with a median age of 70 years (IQR: 62–77), compared to 68 years (IQR: 59–75) in the isolated ostial LAD PCI group (*p* = 0.036). The sex distribution was similar between groups, with females comprising 26.6% of the multivessel PCI group and 28.6% of the isolated ostial LAD group (*p* = 0.619). At a median follow-up of 756 days (IQR: 433–1165), the incidence of MACE was comparable between groups, occurring in 28.4% of the multivessel PCI group versus 25.4% in the isolated ostial LAD PCI group (adjusted HR 1.04; 95% CI 0.73–1.48; *p* = 0.81).

**Conclusion:**

In patients undergoing ostial LAD PCI, treatment of an additional vessel did not increase the risk of MACE in long-term follow-up.

## Highlights

• Outcomes of isolated ostial LAD PCI and multivessel PCI including ostial LAD were directly compared.
• Multivessel PCI including ostial LAD treatment did not increase long-term MACE.
• Rates of MI, stroke, mortality, and repeat revascularization were similar between groups.

## Graphical abstract

**Figure:**
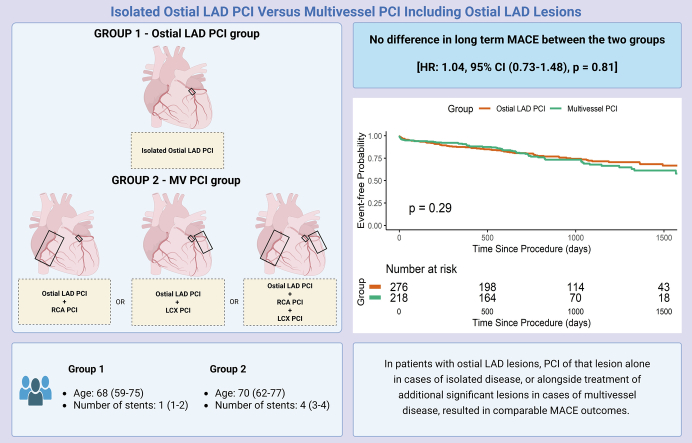

## 1 Introduction

Ostial lesions of the left anterior descending (LAD) coronary artery represent a critical target for revascularization because the LAD supplies blood to a significant portion of the myocardium [1]. Several studies have investigated whether percutaneous coronary intervention (PCI) of ostial LAD artery lesions carries a higher risk of adverse outcomes compared with PCI in other coronary segments [2], [3], [4]. Most findings in the drug-eluting stent (DES) era suggest comparable clinical outcomes; however, it has also been reported that restenosis rates are higher in the ostial LAD group compared with non-ostial lesions [2], [3], [4]. In the broader context of coronary artery disease, multivessel (MV) involvement is common and may complicate both procedural planning and long-term prognosis [5]. Previous studies have reported conflicting results, with some showing similar outcomes between multivessel and single-vessel disease, while others have demonstrated higher mortality and adverse event rates in patients with multivessel disease [5], [6], [7], [8]. Therefore, the impact of treating additional vessels in patients undergoing PCI for an ostial LAD lesion on long-term clinical outcomes, particularly major adverse cardiovascular events (MACE), remains unclear. Understanding whether additional vessel interventions confer a significant benefit or risk is crucial for optimizing treatment strategies.

This study aims to evaluate differences in MACE rates between two patient groups: those who underwent PCI for an ostial LAD lesion alone, and those who underwent PCI for an ostial LAD lesion plus at least one additional significant lesion in another coronary artery.

## 2 Methods

### 2.1 Study design and patient population

This study was a single-center retrospective cohort study. All consecutive patients treated for significant LAD disease between June 2017 and September 2023 were screened for eligibility.

Patients with a significant ostial LAD stenosis (defined as ≥50% stenosis within 5 mm of the ostium of LAD) who underwent stent implantation were eligible for the study. Multivessel disease was defined as significant ostial LAD stenosis with concomitant significant stenosis (>70%) in 1 or more major coronary arteries of 2.5 mm diameter or more or stenosis <70% with functional ischemia. Patients with a history of coronary artery bypass grafting (CABG), concomitant left main (LM) disease (defined as ≥50% stenosis), concomitant ostial left circumflex (LCX) disease (defined as ≥50% stenosis), or in-stent restenosis of a previous ostial LAD stent were excluded. Patients with ostial LAD PCI and multivessel coronary artery disease left untreated, including chronic total occlusions, were also excluded. Two cardiologists reviewed (EN, WC) all coronary angiogram images.

**Fig. 1:**
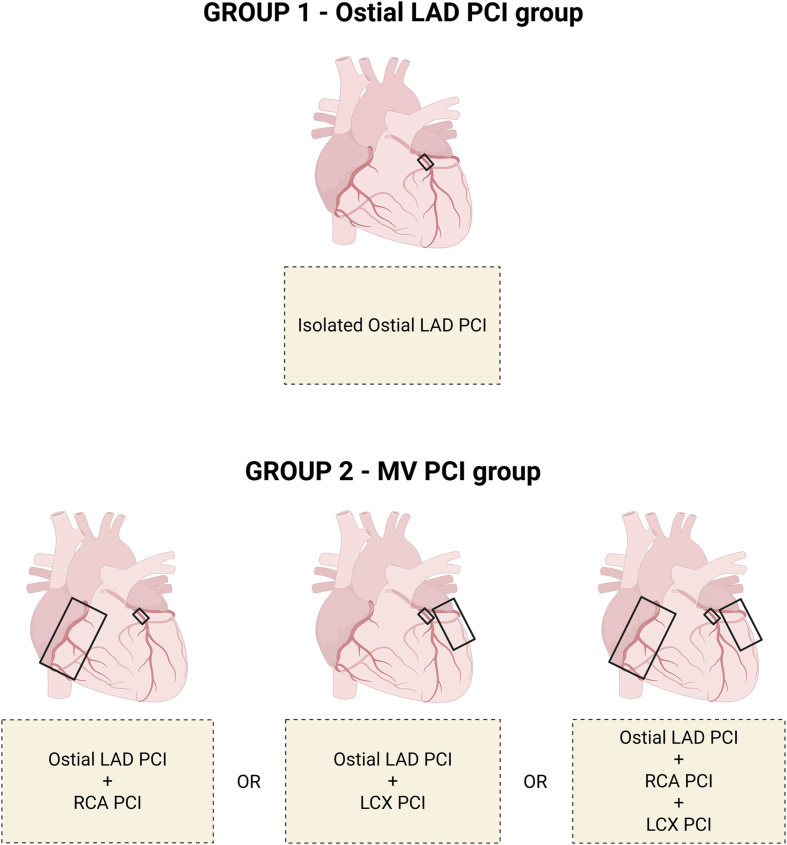
Study groups. Schematic representation of the study groups. Group 1: isolated ostial LAD PCI. Group 2: MV PCI including ostial LAD PCI, consisting of ostial LAD PCI + RCA PCI, ostial LAD PCI + LCX PCI, or ostial LAD PCI + RCA PCI + LCX PCI. LAD, left anterior descending coronary artery; LCX, left circumflex coronary artery; MV, multivessel; PCI, percutaneous coronary intervention; RCA, right coronary artery.

Patients were divided into two groups based on the revascularization approach: isolated ostial LAD PCI and MV PCI (ostial LAD PCI combined with PCI of at least one additional significant lesion in another coronary artery). The distribution of groups is illustrated in Fig. 1. The study was not subject to the Dutch Research on Human Subjects Act, and the need for informed consent was waived.

### 2.2 Endpoints

The primary endpoint was MACE, a composite of all-cause mortality, myocardial infarction (MI), unplanned repeat revascularization and cerebrovascular accident (CVA). Secondary endpoints included the individual components of the primary outcome. Clinical events were defined according to the Academic Research Consortium [9]. MI was reported in accordance with the fourth universal definition of MI [10].

### 2.3 Data collection

Baseline characteristics and procedural details were obtained from the electronic health records at Erasmus University Medical Center. Information on follow-up and outcomes was gathered via electronic health records, written questionnaires completed by patients, or telephone interviews. Time of censoring was defined as the last moment of telephone contact or the last completion of a questionnaire, or, in the absence thereof, the last moment of contact as described in the electronic health records. All collected data were securely stored in a password-protected database, with access restricted to authorized individuals only.

### 2.4 Statistical analysis

Continuous variables were expressed as medians (first and third quartiles) while categorical data were expressed as counts and percentages. Categorical variables were tested with the use of the chi-square or Fisher-Freeman-Halton exact test if expected cell counts were less than five. For continuous variables, comparisons were made using the Mann-Whitney *U* test. The occurrence of study endpoints over time was analyzed using the Kaplan–Meier method and statistical differences between curves were assessed by the log-rank test. Censoring occurred at the last follow-up date available if end point-free, as described above. A univariable Cox regression analysis was conducted to compare clinical outcomes between the isolated ostial LAD PCI and MV PCI groups. Subsequently, multivariable Cox proportional hazards models were applied. All clinical, procedural and angiographic variables were tested in the univariable analysis. Covariates were determined on the basis of historical relevance or a relationship with outcome in the univariable Cox proportional hazards models (*p* value <0.1). One variable per 10 events was taken into account to avoid overfitting of the model. The following variables were included in each model: for MACE, age, diabetes mellitus, hypertension, calcification, prior CVA, chronic obstructive pulmonary disease (COPD), crossover stenting, prior MI, and prior PCI; for all-cause mortality, age, diabetes mellitus, hypertension, calcification, prior CVA, COPD, and crossover stenting; for repeat revascularization, prior PCI, hypertension, and prior MI; and for MI, age. The smaller number of variables included in the models for all-cause mortality and repeat revascularization was due to the limited number of events for these outcomes. Due to the limited number of events, only univariable analysis was performed for CVA. The results of Cox regression were presented as hazard ratio (HR) with 95% confidence intervals (CIs). A 2-sided *p*-value <0.05 was considered significant. The *p*-values were not adjusted for multiple testing. Data were analyzed using both SPSS version 28.0.1.0 and R version 4.4.2.

## 3 Results

**Fig. 2:**
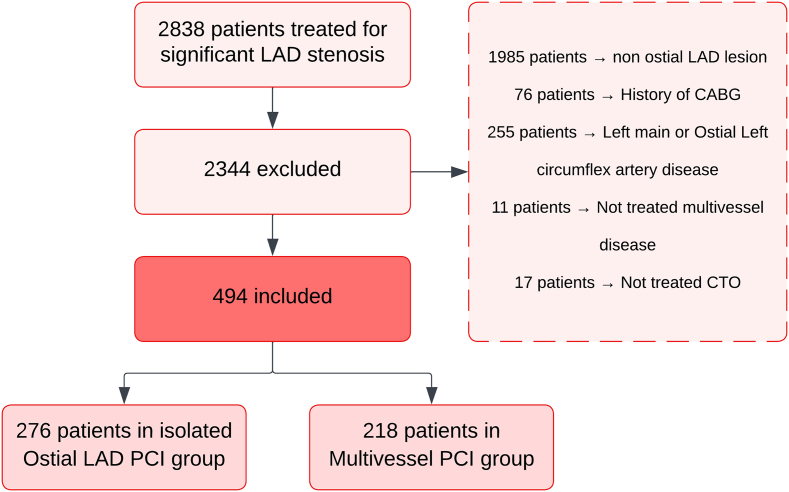
Study flowchart. LAD, left anterior descending; CABG, coronary artery bypass grafting; CTO, chronic total occlusion; PCI, percutaneous coronary intervention.

We included 494 consecutive patients who underwent PCI of ostial LAD alone or alongside with other coronary arteries. Of those, 276 patients were included in the isolated ostial LAD PCI group and 218 in the MV PCI group. The selection process of patients is illustrated in the flowchart (Fig. 2).

### 3.1 Baseline characteristics

Patients in the MV PCI group were older, with a median age of 70 years (IQR: 62–77), compared to 68 years (IQR: 59–75) in the isolated ostial LAD PCI group (*p* = 0.036). Female sex was 28.6% in the isolated ostial LAD PCI group versus 26.6% in the MV PCI group (*p* = 0.619). Clinical presentation was also similar between groups. Other baseline characteristics were similar and are presented in Table 1.

**Table 1:** Baseline Characteristics.

	Ostial LAD PCI	Multivessel PCI	P Value
	(N = 276)	(N = 218)	
Age	68 (59–75)	70 (62–77)	0.036
Female sex	79 (28.6%)	58 (26.6%)	0.619
Presentation			0.237
CCS	81/276 (29.3%)	80/218 (36.7%)	
UA	20/276 (7.2%)	16/218 (7.3%)	
NSTEMI	91/276 (33.0%)	71/218 (32.6%)	
STEMI	84/276 (30.4%)	51/218 (23.4%)	
Smoker	60/259 (23.2%)	39/214 (18.2%)	0.189
Diabetes	56/276 (20.3%)	54/218 (24.8%)	0.235
Hyperlipidemia	117/263 (44.5%)	100/209 (47.8%)	0.467
Hypertension	143/268 (53.4%)	133/215 (61.9%)	0.061
Family history of CVD	69/259 (26.6%)	63/208 (30.3%)	0.384
Medical history			
Stroke	20/276 (7.2%)	20/218 (9.2%)	0.435
PCI	47/275 (17.1%)	48/218 (22.0%)	0.168
MI	37/274 (13.5%)	42/218 (19.3%)	0.084
AF	38/272 (14.0%)	18/214 (8.4%)	0.057
COPD	15/272 (5.5%)	11/217 (5.1%)	0.827
Heart failure			0.932
HFpEF	238/265 (89.8%)	189/211 (89.6%)	
HFmrEF	27/265 (10.2%)	22/211 (10.4%)	

Data are presented as median and Q1 to Q3, or as count and percentage. AF, atrial fibrillation or flutter; CCS, chronic coronary syndrome; COPD, chronic obstructive pulmonary disease; CVD, cardiovascular disease; HFmrEF, heart failure with mildly reduced ejection fraction; HFpEF, heart failure with preserved ejection fraction; MI, myocardial infarction; NSTEMI, non-ST-segment elevation myocardial infarction; PCI, percutaneous coronary intervention; STEMI, ST-segment elevation myocardial infarction; UA, unstable angina.

### 3.2 Procedural characteristics

Mechanical circulatory support was required in 6.6% of patients in the isolated ostial LAD group and in 5.6% of patients in the MV PCI group (*p* = 0.628). Among those patients, bailout support was needed in 55.6% of patients in isolated ostial LAD PCI group and 16.7% of patients in MV PCI group (*p* = 0.058). Intra-aortic balloon pump as bailout support was more frequently used in the isolated ostial LAD PCI group compared to the MV PCI group (66.6% vs. 16.7%, *p* = 0.023). The median number of stents used was 1 (IQR: 1–2) in the isolated ostial LAD PCI group and 4 (IQR: 3–4) in the MV PCI group. In the MV PCI group, the median number of vessels treated was 2 (IQR: 2–3) and 24.3% underwent three-vessel PCI while only 5% of cases involved staged MV PCI (Table 2).

**Table 2:** Procedural characteristics.

	Ostial LAD PCI	Multivessel PCI	P Value
	(N = 276)	(N = 218)	
French size			0.259
6Fr	273 (98.9%)	218 (100%)	
7Fr	3 (1.1%)	0 (0%)	
Access site			0.244
Femoral	10 (3.6%)	3 (1.4%)	
Radial	263 (95.3%)	211 (96.8%)	
Both	3 (1.1%)	4 (1.8%)	
Plaque modification			
IVL	8 (2.9%)	3 (1.4%)	0.361
Atherectomy:			0.216
Orbital	6 (2.2%)	5 (2.3%)	
Rotational	5 (1.8%)	10 (4.6%)	
Intracoronary imaging			0.516
IVUS	97 (35.1%)	69 (31.7%)	
OCT	35 (12.7%)	24 (11.0%)	
FFR	46 (16.7%)	34 (15.6%)	0.748
Mechanical support	18/272 (6.6%)	12/216 (5.6%)	0.628
Planned	8/18 (44.4%)	10/12 (83.3%)	0.058
Bailout	10/18 (55.6%)	2/12 (16.7%)	
IABP	12/18 (66.6%)	2/12 (16.7%)	0.023
ECMO	3/18 (16.7%)	3/12 (25.0%)	>0.99
Impella	4/18 (22.2%)	8/12 (66.7%)	0.111
Dominance			0.717
Right	240 (87.0%)	194 (89.0%)	
Left	32 (11.6%)	20 (9.2%)	
Co-dominance	4 (1.4%)	4 (1.8%)	
Calcification			0.148
None	208 (75.4%)	154 (70.6%)	
Moderate	53 (19.2%)	46 (21.1%)	
Severe	15 (5.4%)	18 (8.3%)	
Crossover Stenting	77 (27.9%)	54 (24.8%)	0.434
Vessels treated	1 (1–1)	2(2–3)	<0.001
Number of stents	1 (1–2)	4 (3–4)	<0.001
RCA	0	128	
LCX	0	143	
RCA and LCX	0	53	

Data are presented as median and Q1 to Q3, or as count and percentage.

ECMO, extracorporeal membrane oxygenation; FFR, fractional flow reserve; Fr, French; IABP, intra-aortic balloon pump; IVL, intravascular lithotripsy; IVUS, intravascular ultrasound; LAD, left anterior descending coronary artery; LCX, left circumflex coronary artery; OCT, optical coherence tomography; PCI, percutaneous coronary intervention; RCA, right coronary artery.

### 3.3 Endpoints

Patients were followed for a median of 756 days (IQR: 433-1165).

**Fig. 3:**
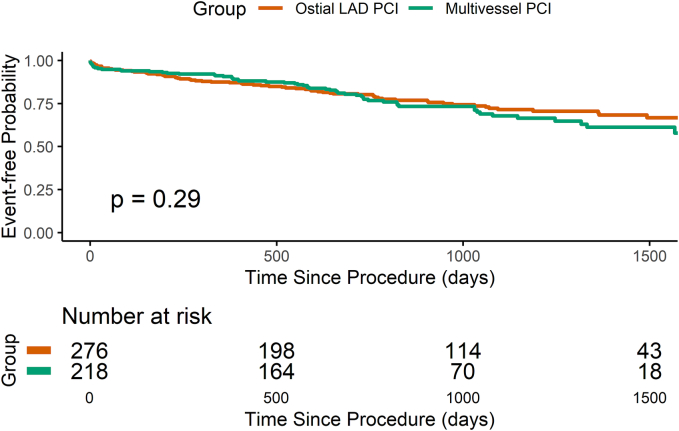
Event-free survival curves for MACE. MACE, major adverse cardiovascular events;

**Fig. 4:**
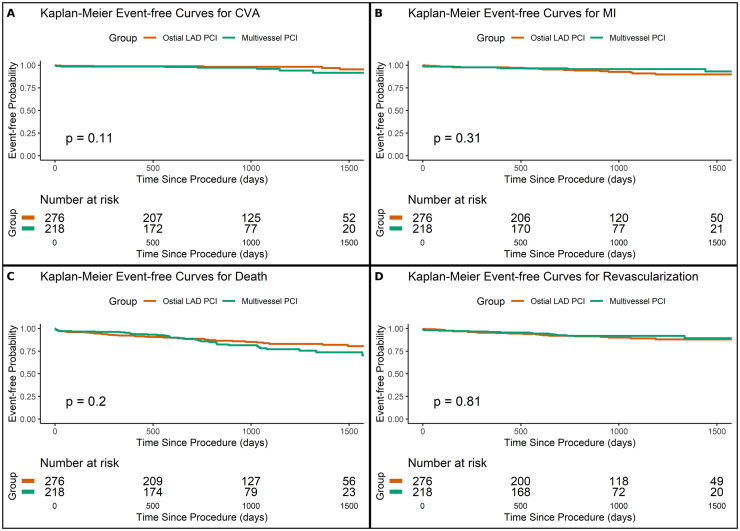
Event-free survival curves for CVA (A), MI (B), death (C) and unplanned repeat revascularization (D). CVA, cerebrovascular accident; MI, myocardial infarction; PCI, percutaneous coronary intervention.

The primary outcome occurred in 62 patients (28.4%) in the MV PCI group and in 70 patients (25.4%) in the isolated ostial LAD PCI group. By univariable analysis, MACE rates did not differ significantly between isolated ostial LAD PCI and MV PCI groups [HR 1.18, 95% CI (0.84–1.67), *p* = 0.33]. These findings remained consistent in the adjusted analysis [HR 1.04, 95% CI (0.73–1.48), *p* = 0.81]. No significant differences were observed between the 2 groups in secondary endpoints of all-cause mortality, MI, unplanned revascularization and CVA in univariable analysis and after multivariable adjustment (Table 3). Fig. 3, Fig. 4 show the time-to-event Kaplan-Meier curves for the primary endpoint and for secondary outcomes between the 2 groups.

**Table 3:** Clinical outcomes in patients undergoing multivessel vs ostial LAD stenting in univariable and multivariable cox regression model.

	Multivessel PCI (n = 218)	Ostial LAD PCI (n = 276)	Unadjusted Hazard ratio (95% CI)	P value	Adjusted Hazard ratio (95% CI)	P value
Primary outcome						
MACE^a^	62 (28.4%)	70 (25.4%)	1.18 (0.84–1.67)	0.33	1.04 (0.73–1.48)	0.81

Secondary outcomes						
CVA	9 (4.1%)	6 (2.2%)	2.34 (0.83–6.63)	0.11		
MI^b^	9 (4.1%)	18 (6.5%)	0.67 (0.30–1.50)	0.33	0.65 (0.29–1.46)	0.30
All-cause mortality^c^	40 (18.3%)	41 (14.9%)	1.36 (0.88–2.11)	0.17	1.19 (0.76–1.87)	0.44
Revascularization^d^	17 (7.8%)	24 (8.7%)	0.85 (0.45–1.60)	0.62	0.74 (0.39–1.39)	0.35

COPD, chronic obstructive pulmonary disease; CVA, cerebrovascular accident; LAD, left anterior descending coronary artery; MACE, major adverse cardiovascular events; MI, myocardial infarction; PCI, percutaneous coronary intervention.

a Covariates used for MACE were age, diabetes mellitus, hypertension, calcification, prior CVA, COPD, crossover stenting, prior MI, prior PCI.

b Covariate used for MI was age.

c Covariates used for all-cause mortality were age, diabetes mellitus, hypertension, calcification, prior CVA, COPD, crossover stenting.

d Covariates used for revascularization were prior PCI, hypertension, prior MI.

## 4 Discussion

To our knowledge, this is the first study to directly compare long-term outcomes between isolated ostial LAD PCI and MV PCI including ostial LAD treatment. We observed no significant differences in MACE, all-cause mortality, MI, unplanned revascularization, or CVA. These findings suggest that when complete revascularization is achieved, outcomes of MV PCI can approximate those of isolated ostial LAD PCI.

Multivessel coronary artery disease, if left untreated, is associated with a worse prognosis, particularly as the extent of atherosclerosis increases [5], [11], [12].

A large registry analysis previously demonstrated that patients undergoing PCI for multivessel coronary artery disease had higher long-term mortality than those for single-vessel disease. Mortality risk was nearly doubled in patients with both diabetes and multivessel disease when compared to patients without diabetes and with single-vessel disease and remained elevated for multivessel disease alone [6]. Similarly, a registry of patients undergoing PCI for chronic total occlusion found worse outcomes in multivessel disease, including higher MACE rates [7]. Both studies did not specifically investigate single-vessel ostial LAD disease. In contrast, our study suggests that when complete revascularization is achieved, outcomes in multivessel PCI can be comparable to those of single-vessel intervention involving the ostial LAD.

In contrast to the above-mentioned studies, there are also studies that show comparable results between multivessel and single-vessel disease. A sub-study of the CADILLAC trial demonstrated that patients with multivessel disease undergoing subsequent PCI of non-infarct-related arteries had survival comparable to patients with single-vessel disease [5]. Similarly, a retrospective analysis of patients younger than 50 years undergoing coronary stent implantation showed that outcomes in multivessel disease were similar to those in single-vessel disease. In that cohort, 50% of single-vessel patients had LAD involvement, whereas LAD was involved in 31.4% of multivessel patients [8]. Our findings extend these observations by showing that, specifically in ostial LAD lesions, outcomes of MV PCI with complete revascularization are comparable to those of isolated ostial LAD PCI.

Previous studies of ostial LAD lesions often compared them with non-ostial LAD or other aorto-ostial lesions, but did not specifically address their role in the setting of multivessel revascularization. Our findings suggest that revascularization of the ostial LAD—a lesion with particularly high ischemic burden—may be the main determinant of outcomes, regardless of whether additional lesions are treated concurrently. In a broader context, it is important to recognize that when comparing outcomes between single-vessel and multivessel disease treatments, the results may largely depend on the specific location of the lesion and which single-vessel is treated. In the bare-metal stent era, ostial LAD PCI was associated with higher restenosis rates compared with non-ostial LAD lesions, despite similar procedural success and in-hospital outcomes [4]. Yamamoto et al. also reported higher cardiac mortality in patients with ostial proximal LAD lesions compared with non-ostial lesions, although differences in MACE did not reach significance [13]. The introduction of DES has substantially improved outcomes. A sub-analysis of the J-Cypher registry showed that 3-year results after sirolimus-eluting stent implantation for ostial LAD lesions were comparable to non-ostial proximal LAD disease [2]. The CAPTAIN study similarly reported equivalent in-hospital MACE rates among LAD-ostial, aorto-ostial, and non-ostial lesions treated with DES [3]. However, all of these analyses were limited to single-vessel disease.

Advances in stent platforms, intravascular imaging, and procedural techniques may explain why procedural complexity may no longer translate into worse outcomes. In the OPTIVUS-Complex PCI study, 1-year outcomes of patients undergoing complex IVUS-guided PCI were similar to those of less complex procedures [14], [15]. In our cohort, imaging guidance was used in nearly half of cases across both groups. Likewise, data from the Mount Sinai Hospital registry showed that adding non-LM PCI to LM complex PCI did not increase MACE risk at 1 year, supporting the concept that complex multivessel PCI can be performed safely with outcomes largely determined by the most complex lesion [16].

In summary, our study directly compares isolated ostial LAD PCI with multivessel PCI including ostial LAD intervention, highlighting the central role of ostial LAD revascularization in determining prognosis and revascularization strategy. These results reflect the evolution of PCI practice, in which modern technologies and complete revascularization strategies have mitigated the risks traditionally associated with procedural complexity.

## 5 Limitations

Firstly, this was an investigation that was conducted at a single-center and clinical events were not adjudicated by an independent committee. Secondly, this was a non-randomized retrospective study and certain selection biases and unknown confounding factors possibly impacting outcomes could not be excluded. Thirdly, the choices regarding treatment strategies were left to the discretion of the operators and were not based on predefined criteria. Finally, this study was conducted at a high-volume academic tertiary care center with broad availability of intravascular imaging, calcium-modification techniques, and mechanical circulatory support. As such, the findings may not be fully generalizable to lower-volume or non-tertiary centers, where operator experience, procedural planning, and access to advanced adjunctive technologies and mechanical support may differ.

## 6 Conclusion

In patients with ostial LAD lesions, PCI of that lesion alone in cases of isolated disease, or alongside treatment of additional significant lesions in cases of multivessel disease, resulted in comparable MACE outcomes.

## Glossary

LAD: Left anterior descending (coronary artery)
PCI: Percutaneous coronary intervention
MV: Multivessel
MACE: Major adverse cardiovascular events
MI: Myocardial infarction
CVA: Cerebrovascular accident (stroke)
DES: Drug-eluting stent

## CRediT authorship contribution statement

**Eleni Ntantou:** Writing – review & editing, Writing – original draft, Visualization, Validation, Resources, Project administration, Methodology, Investigation, Formal analysis, Data curation, Conceptualization. **William Camilleri:** Writing – review & editing, Visualization, Validation, Resources, Project administration, Methodology, Investigation, Data curation, Conceptualization. **Joost Daemen:** Writing – review & editing, Methodology, Investigation, Conceptualization. **Roberto Diletti:** Writing – review & editing, Methodology, Investigation, Conceptualization. **Jeroen Wilschut:** Writing – review & editing, Methodology, Investigation, Conceptualization. **Isabella Kardys:** Writing – review & editing, Validation, Formal analysis. **Rutger-Jan Nuis:** Writing – review & editing, Methodology, Investigation, Conceptualization. **Nicolas M. van Mieghem:** Writing – review & editing, Methodology, Investigation, Conceptualization. **Wijnand K. den Dekker:** Writing – review & editing, Supervision, Project administration, Methodology, Investigation, Data curation, Conceptualization.

## Funding and disclosures

This research did not receive any specific grant from funding agencies in the public, commercial, or not-for-profit sectors.

## Ethical statement

The study was not subject to the Dutch Research on Humans Subjects Act, and the need for informed consent was waived.

## Declaration of competing interest

The authors declare that they have no known competing financial interests or personal relationships that could have appeared to influence the work reported in this paper.
